# Anti-HLA Class I alloantibodies in platelet transfusion refractoriness: From mechanisms and determinants to therapeutic prospects

**DOI:** 10.3389/fimmu.2023.1125367

**Published:** 2023-02-09

**Authors:** Adèle Couvidou, Gabriel Rojas-Jiménez, Arnaud Dupuis, Blandine Maître

**Affiliations:** ^1^ UMR_S1255, INSERM, Strasbourg, France; ^2^ Etablissement Français du Sang-Grand Est, Strasbourg, France; ^3^ Fédération de Médecine Translationnelle de Strasbourg (FMTS), Strasbourg, France; ^4^ Université de Strasbourg, Strasbourg, France

**Keywords:** HLA class I, alloantibodies, transfusion, platelets, refractoriness (platelets)

## Abstract

Patients with hematological disorders and severe thrombocytopenia require extensive and iterative platelet transfusion support. In these patients, platelet transfusion refractoriness represents a serious adverse transfusion event with major outcomes for patient care. Recipient alloantibodies against the donor HLA Class I antigens expressed at the cell surface of platelets result in a rapid removal of transfused platelets from the circulation and thus, therapeutic and prophylactic transfusion failure leading to a major bleeding risk. In this case, the only way to support the patient relies on the selection of HLA Class I compatible platelets, an approach restricted by the limited number of HLA-typed donors available and the difficulty of meeting the demand in an emergency. However, not all patients with anti-HLA Class I antibodies develop refractoriness to platelet transfusions, raising the question of the intrinsic characteristics of the antibodies and the immune-mediated mechanisms of platelet clearance associated with a refractory state. In this review, we examine the current challenges in platelet transfusion refractoriness and detail the key features of the antibodies involved that should be considered. Finally, we also provide an overview of future therapeutic strategies.

## Introduction

The management of patients with severe thrombocytopenia may require extensive and iterative platelet transfusion support. A serious complication of these multiple transfusions is the platelet transfusion refractory state (PTR), characterized by ineffective transfusion efficiency. Immune and non-immune causes can lead to PTR. Non-immune factors, which account for 80% of PTR cases, include all causes of platelet hyperconsumption such as acute inflammatory conditions, e.g. infection, splenomegaly or disseminated intravascular coagulation (1). On the other hand, immune factors, i.e. adaptive immune response through humoral and cellular components, which play a role in approximately 20% of patients with PTR, include antibodies directed against HLA Class I (HLA-I) molecules and/or against human platelets antigens (HPA). Nonetheless, anti-HLA-I antibodies are considered to be the main cause of immune-mediated PTR. In both cases, PTR is associated with serious outcomes, including longer hospital stays (2), higher treatment costs, excessive use of labile blood products, and increased risk of death (3, 4).

The anti-HLA-I antibodies associated with PTR may lead to the clearance of transfused platelets, resulting in ineffective platelet transfusions to prevent and/or stop bleeding. Immune PTR can potentially be circumvented by using HLA I-matched platelets, i.e. perfectly HLA I-matched platelets between donor and recipient, platelets carrying only antigens against which the recipient has not developed antibodies (permissive antigens) (5, 6) or crossmatched platelets (7). However, finding a suitable donor can be difficult, especially in the case of a highly immunized patient. Indeed, this method necessitates knowledge of the recipient’s HLA phenotype and access to a large database of donors with a known HLA phenotype. This requires a significant amount of time and workload, sometimes incompatible with the urgency of transfusion support. According to the “Trial to reduce alloimmunization to platelets” (TRAP) study, the presence of anti-HLA-I antibodies in the recipient is correlated to refractoriness. Platelet concentrate preparation processes, such as leukoreduction or platelet concentrate storage time, which impact alloantibody production, are parameters that indirectly influence the incidence of PTR (8). Nevertheless, not all patients with anti-HLA-I antibodies develop PTR upon platelet transfusions, raising the question of the antibodies’ intrinsic features associated with PTR. Identifying the mechanisms by which platelets are removed from the circulation and determining the characteristics of anti-HLA-I antibodies associated with PTR would improve the management of patients who require transfusion support.

In this review, we describe the clinical criteria for PTR and outline the main features of HLA-I antibodies which could be considered in the context of transfusion deadlock. Finally, we discuss the current challenges of PTR and provide a brief perspective on future therapeutic strategies.

## Definition and diagnosis criteria of platelet transfusion refractoriness

In non-bleeding patients with thrombocytopenia, PTR is defined as two repeated transfusion failures with, ABO-identical platelets and a quantity adapted to the patient's weight. In addition, in some countries, the notion of “fresh” platelets (i.e. less than 3 days of storage) is included in this definition, which reinforces the stringency of the criteria for concluding a PTR. To assess proper response to platelet transfusion, one must follow platelet count increments at 60 minutes and 24h post transfusion. The corrected count increment (CCI) is widely used to assess the post-transfusion platelet increment. The CCI is derived from the following equation: ((Platelet count after transfusion – platelet count before transfusion) x body surface area (m^2^) x 100)/(Number of platelets transfused × 10^11^).

There is no consensus yet on the threshold of the CCI value indicating a PTR. The TRAP study defined refractoriness as a CCI value < 5000 (9) but a CCI <7500 is also an accepted value to indicate a PTR (10). Studies have shown that platelets need at least 60 minutes to equilibrate in the intravascular space, which is why platelet counts should not be measured before 1 hour after the end of the transfusion (11). However, several groups have reported the relevance of taking the platelet count at 10 min for practical reasons and because the 10 min count might be as informative as the platelet count at 1h (12).

Theoretically, the calculation of the CCI at 1h and at 24h can be used to discriminate immune from non-immune PTR. In non-immune PTR, the platelet count rises 1 hour after platelet transfusion but falls again after 24 hours, whereas in immune PTR, the transfused platelets are rapidly cleared from the circulation and there is no increase in platelet count 1 hour after transfusion (13, 14).

Once refractoriness has been confirmed, non-immune causes must be explored and ruled out in order to ascertain an immune PTR. Bleedings, infections, inflammatory conditions, or drug interference should be considered before testing the presence of anti-HLA-I antibodies and requesting HLA-matched platelets (15). However, in patients with hematological disorders, immune and non-immune factors are often not mutually exclusive and may be present simultaneously (6, 16, 17). All the criteria required for defining a refractory state are very strict, and not necessarily compatible with daily clinical practice. In particular, CCI is of no value in thrombocytopenic patients who bleed. In this situation, the only way to ascertain the efficiency of platelet transfusion is to observe the arrest of bleeding. Depending on the patient’s pathology and the country in which the clinical study is conducted, the occurrence of PTR can range from 10% to 49% (18). This wide range of incidence possibly reflects the variability due to the challenge of diagnosing PTR.

## Mechanisms leading to clearance of transfused platelets during an immune PTR

**Figure 1:**
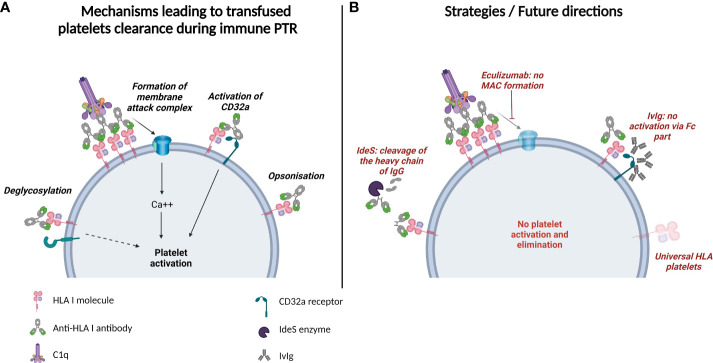
Mechanisms of platelet elimination and prospective therapies in HLA Class I-alloantibody-mediated PTR. **(A)** Potential mechanisms leading to transfused platelets clearance during immune platelet transfusion refractoriness. Anti HLA Class I-alloantibodies can opsonize platelets or lead to their activation *via* deglycosylation, formation of membrane attack complex or CD32a receptor mobilization. **(B)** Strategies/Future directions in platelet transfusion refractoriness: cleavage of the heavy chain of IgGs by IdeS enzyme, inhibition of the membrane attack complex formation with Eculizumab, inhibition of Fc receptor mobilization with IvIg, and transfusion of universal HLA platelets that do not express HLA molecules on the surface.

Anti-HLA-I alloantibodies can lead to clearance of transfused platelets. Several mechanisms for antibody-mediated platelet clearance have been described (**Figure 1A**):

### Opsonisation

The first function of antibodies is to act as opsonins to promote phagocytosis of sensitized platelets (19). Antibodies are fixed to the target, leading to activation of Fc receptors and phagocytosis. Anti-HLA-I antibodies or auto-antibodies directed against platelets, in case of immune thrombocytopenia (characterized by an isolated thrombocytopenia), appear to be partially caused by IgG-mediated platelet destruction in the spleen (20). Moreover, internalization of platelets with high HLA-I density by macrophages has been shown to be significantly increased in contrast to platelets coming from patients with low HLA-I density at the surface. Thus, the degree of antibody opsonisation and antigen expression directly correlates with antibody-mediated internalization of platelets by macrophages, suggesting that platelets expressing fewer antigens could be used in priority to treat refractory patients (21).

### Activation

Platelet activation mediated by anti-HLA-I antibodies could partially explain immune platelet clearance during refractoriness. Some antibodies directed against HLA-I molecules have been reported to induce platelet activation (22, 23). Depending on both the antibodies and the recognized epitopes, two mechanisms have been described. First, anti-HLA-I antibodies can mobilize the FcγRIIa/CD32a expressed on the cell surface of platelets, which leads to platelet activation. FcγRIIa is a low affinity receptor for IgG subclasses. It bears an intracellular immunoreceptor tyrosine-based activation motif (ITAM) that mediates cell activation upon its crosslinking by IgG-immune complexes (24). HLA-I antibodies can initiate the signaling cascade, leading to ITAM phosphorylation and consequent platelet activation (22). Using human monoclonal antibodies that recognize different epitopes of HLA-I on platelets, the same group showed that blocking the classical pathway of the complement, by using anti-C1q or anti-C5 (Eculizumab) antibodies, inhibited to some extent the level of platelet activation. Finally, the activation induced by anti-HLA-I antibodies can be fully abrogated by the inhibition of the CD32a receptor with monoclonal antibody IV.3 in combination with complement classical pathway inhibitor Eculizumab, meaning that both FcyRIIa and complement activation pathways can act in synergy (23).

### Desialylation

Antibody-mediated platelet clearance can also be explained by deglycosylation. In immune thrombocytopenia, anti-GPIbα antibodies cross-link GPIbα subunits, initiating activation and signaling. The activation of platelets conducts to granule secretion, after which released CD62P and NEU1 desialylate surface sialic acid residues, particularly on GPIbα. The removal of the bulky terminal sialic residues facilitates assembly of the GPIb complex and also triggers activation, thus forming an activation loop (25). Once desialylated, platelets are trapped in the liver where they can be cleared by Kupffer cells, a subset of liver professional phagocytes. A collaboration between Ashwell Morell receptors and macrophage galactose lectins, both expressed by Kupffer cells, has been described to eliminate efficiently desialylated platelets (26). More recently, the C-type lectin receptor, CLEC4F, expressed specifically on Kupffer cells, has been reported to recognize desialylated glycans and to participate in the clearance of aged platelets (27). The question of potential desialylation of platelets during PTR is still open.

## Intrinsic features of anti-HLA-I antibodies

As not all HLA-I antibodies induce platelet refractoriness, analysis of antibodies’ features can be relevant to identify those capable of pathogenicity. Here we describe the main features of HLA-I antibodies that should be taken into consideration in a PTR context.

### Isotypes and IgG subclass

The isotypes involved in PTR are predominantly IgG (28). IgGs, the major immunoglobulins in serum, are divided into four subclasses: IgG1, IgG2, IgG3 and IgG4. The differences between each subclass lie in the amino acid composition of their heavy chain and have a decisive impact on the hinge region as well as in their relative abundance. Each subclass has therefore a particular profile, particularly in terms of ability to activate the complement cascade, with IgG1 and IgG3 being more efficient in complement activation than IgG2 and IgG4, and their affinity for the Fc receptors (FcR), meaning that anti-HLA-I antibody subclasses could correlate with distinct effects on platelets (29). Moreover, a study performed in baboons suggested that the subclasses could influence the location of platelet sequestration. They showed that platelets were sequestered in the spleen when the anti-platelet antibody was IgG1, whereas IgG2 led mostly to liver elimination (30). A link between anti-HLA-I antibody subclasses and allograft outcome has also been studied, suggesting a correlation between complement fixation and allograft failure (31). All of these observations indicate that it might be useful to consider the HLA-I antibody subclass when facing an immune PTR.

### Glycosylation

IgG glycans are essential for the maintenance of functional structure and can evolve depending on time, age, disease, or environmental factors. The glycosylation of antibodies affects their affinity for FcR and their ability to mobilize complement (32) thus potentially modulating their ability to activate platelets.

Analysis of sera from alloimmunized patients showed that Fc glycosylation of anti-HLA-I antibodies is highly variable. Using recombinant glycoengineered anti-HLA monoclonal antibodies with variable Fc glycosylation, Van Osch et al. showed that galactosylation of human anti-platelet IgG enhances complement activation by promoting hexamerization of hIgG1 which constitutes an optimal platform for C1q to bind (33). Furthermore, Kapur et al. showed that anti-HLA-I IgG, collected from alloimmunized patients in PTR or from patients with neonatal thrombocytopenia, are more galactosylated than total serum IgG1 levels, but they do not present different levels of sialylation and fucosylation. Modulation of galactosylation can affect IgG clearance *in vivo* and predisposes them for further modification by the addition of sialic acid. By contrast, anti-human platelet antigens antibodies are less fucosylated than total serum IgG1, leading to enhanced phagocytosis of IgG-binded platelets and increased disease severity (34). Accordingly, HLA-specific afucosylated IgG1 have been proposed to be a potential predictor of antibody pathogenicity (35), illustrating the importance of sugars in antibody functions.

### Antigen specificity

As analyzed by Wang et al., in a 204-patient cohort, alloantibodies were directed towards the most prevalent HLA-I A and B molecules in that population (36). WIM8E5, an anti-HLA-I monoclonal antibody that recognizes an epitope on all HLA-A antigens, except for HLA-A3 and HLA-A32 (and reduced binding to HLA-A2), has been suggested to fix complement depending on the number of epitopes available for it; however, it remains to be determined if the identity of these epitopes matters, or just the number of binding sites on HLA-I available on platelets for a given antibody (23).

It has also been proposed that a higher diversity in the antibody specificity correlates with a higher level of HLA-I antibodies as detected by screening. It is likely that the greater the range of specificities, the higher the probability that an antibody will recognize HLA-I antigens on transfused platelets (37). This scenario becomes particularly relevant in multi-transfused (i.e. oncohematologic) patients where multiple exposures could potentially boost titers of specific anti-HLA-I antibodies, as well as elicit antibody responses to new alloantigens (38).

It is possible that the presence of anti-idiotypic antibodies directed against the variable regions of anti-HLA-I antibodies can regulate the latter’s abundance (39). It remains to be studied if the anti-idiotypic antibodies recognize preferentially some V regions in anti-HLA-I with determined specificities for particular HLA antigens.

## Strategies/Future directions

Since the expression of HLA-I molecules on the surface of platelets and their recognition by alloantibodies are responsible for the majority of immune PTR, future strategies to avoid transfusion refractoriness focus on either reducing the expression of HLA antigens or modulating the effects of antibodies (**Figure 1B**).

### Universal HLA-I platelets

Platelet refractoriness could be overcome by transfusing platelets devoid of any HLA-I molecules. The first attempt to remove, or at least decrease, HLA antigens platelet expression was based on chemical modification. Acid treatment of human cells has been reported to eliminate the antigenicity of class I MHC molecules without significant cell death (40). Based on this discovery, several methods for preparing HLA-I depleted platelets have been reported. Acid treatment has been proven to reduce HLA-I molecules expression (41) and the subsequent HLA-antibody-mediated phagocytosis (42). Interestingly, proteomic analyses showed that the changes of platelet proteins after treatment with citric acid were functionally safe (43). However, despite the description of the absence of negative effects on proteins involved in coagulation and haemostasis, these results still need to be confirmed by functional tests. This approach is therefore attractive because it could theoretically be suitable for mass production at a reasonable cost. However, no method has yet been approved for clinical use and research is still ongoing in this field.

A promising but challenging alternative to the chemical treatment could be the use of HLA-I-deficient platelets generated *in vitro*. Different methods of *in vitro* platelet production have been reported, which differ in the source of the stem cells: induced pluripotent stem cells (iPSCs) or hematopoietic progenitors (CD34^+^ cells). Each of these offers advantages and disadvantages for the development of a transfusion product (44). To be expressed on the surface of cells, HLA-I molecules must form a heterodimer between a heavy chain and a light chain, the β2-microglobulin. The strategy used so far to suppress the expression of HLA-I molecules relies on the deletion of the β2-microglobulin gene, consequently leading to the absence of HLA-I molecules on the cell surface. HLA-I deficient platelets produced from iPSCs have been reported to successfully circulate in an alloimmune PTR model of mice reconstituted with human NK cells (45). More recently, results from the first clinical trial of *ex vivo-*generated platelets show that it is a safe product, however, it remains restricted to autologous and therefore individualized *ex vivo* platelet production (46). Thus, optimization is necessary before cultured platelets can become a plausible alternative to the HLA-compatible blood product.

### Targeting the complement pathway

Based on the data suggesting the involvement of complement in platelet HLA-I antibodies-related activation, a pilot study has been designed to assess the efficiency of eculizumab, a monoclonal antibody that inhibits C5 complement component, in platelet transfusion refractoriness (47). In this preliminary study, administration of eculizumab allows some patients to overcome PTR despite their transfusion with HLA-incompatible platelet concentrates. A clinical trial with a larger cohort should provide definitive conclusions concerning this strategy. However, it is important to keep in mind the high cost of such a treatment, which, even if it proves to be effective, will be restricted to real transfusion deadlocks but will not allow the handling of all PTR.

### Repurpose of antineoplastic drugs

Drugs against underlying pathologies common in PTR patients may help in their management when transfusion with HLA-compatible products is not feasible. Rituximab, a monoclonal antibody directed against CD20 expressed on B cells, has been reported to increment platelet count in refractory patients with aplastic anemia or myelodysplatic syndrome (48, 49). Daratumumab, an anti-CD38 monoclonal antibody common in myeloma treatment, has also proven to increase platelet transfusion efficiency in refractory patients (50). However, at least for rituximab, two response profiles were identified: an early and transient response, or a late and continuous response (48). Interestingly, Bortezomib, a specific inhibitor of 26S proteasome, induced a drastic decrease in the amount of anti-HLA-I antibodies in the serum of a patient treated for multiple myeloma, resulting in clinical response to transfusion from random donors (51). In addition, several studies describe an impact of these drugs on platelet count which means that these considerations should be taken into account in the design of future pharmacological strategies to manage refractoriness to platelet transfusions (52, 53). These different approaches seem promising but should be validated with a larger cohort of patients.

### Intravenous immunoglobulin and plasma exchange

One strategy used in antibody-related disease lies on the modulation of antibodies themselves. Intravenous immunoglobulin (IvIg) therapy, defined as the use of a combination of antibodies obtained from healthy human donors, or plasma exchange (PE) have been reported as a successful treatment for PTR patients (54, 55). The clinical benefit of IvIg treatment could be due to blockage of FcγR expressed by the reticuloendothelial system, where opsonized platelets would not be cleared anymore by these cells, and also due to modulation of antibodies’ effects, i.e. interference with the complement or regulation of different FcR (56). *Samuelsson et al.* proposed that the IvIg anti-inflammatory activity is mediated by upregulation of the inhibitory Fc receptor, FcγRIIb, as IvIg has no protective effects on a mouse model of immune thrombocytopenia lacking this receptor (57). Another interesting role for saturation of FcRn could be the increased elimination of circulating pathogenic antibodies. FcRn are MHC-class I like molecules expressed by leukocytes and endothelial cells, which control the half-life of IgG and albumin. FcRn can form a ternary complex on an IgG Fc scaffold under acidic condition, which protects IgGs from endothelial catabolism. However, Crow et al. have suggested that the mechanism of IvIg action is FcRn-independent, as IvIg improved platelet count in a murine model of ITP in FcRn-deficient mice comparable to WT mice (58).

Furthermore, evidence of its benefit in immune-mediated PTR is still lacking and a large clinical study should be conducted to draw firm conclusions about its potential efficiency in this particular context.

### IdeS

IgG-degrading enzyme from *S. pyogenes* (IdeS) is a cysteine protease that cleaves the heavy chain of IgG, generating one F(ab’)2 fragment and two Fc fragments (59). Since IdeS can selectively and rapidly neutralize the Fc-mediated effector function of human IgG, it is a promising new therapy for treating IgG-driven disorders. Recently, an elegant study describing the use of IdeS in a platelet immune disorder has been reported. A recombinant protein with the N-terminus of IdeS and the C-terminus of a single-chain variable fragment from a CD32a antibody was generated. This protein can bind specifically to all cells expressing CD32a, including platelets, and it can neutralize anti-platelet antibodies. Interestingly, platelets with surface-bound IgG-degrading enzymes are protected from clearance in murine models of immune thrombocytopenia (60). This technology could represent a very promising strategy to avoid or alleviate PTR in the future.

## Conclusion

Refractoriness to platelet transfusions remains a major complication, especially for patients requiring chronic transfusion support. Alloantibodies directed towards HLA-I rapidly recognize and eliminate transfused platelets from the recipient’s circulation. Some of these antibodies do not affect transfusion yield, raising questions about the determinants of anti-HLA-I pathogenicity. Further studies are necessary to shed light upon the anti-HLA-I antibodies’ capacity to eliminate transfused platelets and the link to their intrinsic properties, in order to potentially develop new therapeutic approaches to prevent, or at least manage, PTR.

## Author contributions

AC and GR-J wrote the first draft of the manuscript. BM, AC and GR-J wrote sections of the manuscript. AD contributed to the diagnosis/definition criteria and the future direction sections. BM coordinated the work and finalize the manuscript. All authors contributed to the article and approved the submitted version.
